# Experimental Study on Cement-Based Materials Modified by Nano-Zinc Oxide and Nano-Zirconia Based on Response Surface Optimization Design

**DOI:** 10.3390/ma18071515

**Published:** 2025-03-27

**Authors:** Hongyin Hu, Fufei Wu, Jiao Chen, Shuangshuang Guan, Peng Qu, Hongqin Zhang, Yuyi Chen, Zirun Xu, Chuanteng Huang, Shuang Pu

**Affiliations:** 1 School of Materials and Architectural Engineering, Guizhou Normal University, Guiyang 550025, China; 2 Guizhou School of Emergency Management, Guizhou Normal University, Guiyang 550025, China; 3 School of Engineering, Zunyi Normal University, Zunyi 563006, China

**Keywords:** nano-zinc oxide, nano-zirconia, response surface analysis, cement-based materials

## Abstract

Using nanomaterials to replace part of cement is one of the effective ways to enhance the performance of cement-based materials. In this study, the response surface analysis method was used to design an experiment. Through tests on the mechanical properties, the coefficient of water saturation, the shrinkage properties, and the high-temperature calcination of cement-based materials, the effects of three factors, namely, the substitution amount of nano-zinc oxide for cement, the substitution amount of nano-zirconia for cement, and the water–cement ratio, on cement-based materials under different conditions were compared and analyzed. The lower limit of the compressive strength of the cement-based materials increased by 88.17%, and the upper limit increased by 15.14% by using nano-zinc oxide and nano-zirconia to replace part of the cement. The compressive strength of cement-based materials with a nano-zinc oxide content in the range of 0.4–0.6% was low because of the low content of CSH. The coefficient of water saturation decreased with an increase in age, and the coefficient of water saturation of high-performance concrete was low. Nano-zirconia had a significant effect on the mass loss of autogenous shrinkage and the mass loss of drying shrinkage. When the substitution amount of nano-zinc oxide was 0.4–0.8%, the mass loss was large. In summary, with its unique microscopic characteristics, nanomaterials could significantly improve the performance of cement-based materials with regards to their mechanical properties, durability, workability, and other aspects.

## 1. Introduction

With the rapid development of the construction industry, the demand for high-performance building materials is increasing. Meanwhile, traditional building materials are close to the limits of their physical and chemical properties in terms of mechanical properties, durability, and functionalization [1]. Therefore, it is necessary to explore new methods to solve the problem of cement-based material enhancements in the process of building construction and further performance improvements. It has been identified that the utilization of nanomaterials as a partial substitute for binders or as a filler material in cement concrete modifies the rheology and the microstructure at the nano scale, significantly improving the mechanical and durability characteristics of cementitious composites [2,3]. Considering the characteristics of nanomaterials, this paper studies the effects of nano-zinc oxide and nano-zirconia on the enhancement effects of cement-based materials and provides a deep understanding of the mechanism of nanomaterials in cement-based materials so as to provide a scientific basis for optimizing the performance of cement-based materials. Therefore, in this project, nanomaterials were added to cement-based materials for modification experiments to reveal the enhancement mechanism of nanomaterials on the properties of cement-based materials.

According to a survey, many scholars at home and abroad have carried out a lot of in-depth research on cement-based materials mixed with nanomaterials and have explored the influence of nanomaterials on the mechanical properties and durability of cement-based materials at the micro and macro levels and have achieved rich research results. Different kinds of nanomaterials play their respective roles in construction engineering. In China, the research on nanomaterials mainly focuses on the synthesis and functionalization of nanostructures and their applications in energy, environmental governance, biomedicine, and other fields. Chinese scientists have made a series of important achievements in nano-carbon materials [4], metal nanoparticles [5], nano-oxides, and so on. Internationally, cement-based materials mainly focus on the single function of traditional cement-based materials, and researchers are committed to the development of materials with various additional properties, such as thermal insulation [6], fire resistance [7], self-cleaning [8], electromagnetic shielding, ion curing [9], and air purification properties [10]. These characteristics not only meet the basic requirements of modern buildings for the high strength and high durability of materials but also promote the multi-functional development of buildings and help to realize the intelligent transformation of buildings. Starting from the functional modification of cement-based materials by nanomaterials, such as nano-silica, nano-carbon dioxide, carbon nanotubes, and graphene oxide, the effects of the characteristics, incorporation methods, and dosage of different nanomaterials on the functional modification performance of cement-based materials have been analyzed. Research institutions in Europe and the United States have achieved remarkable results in the fields of self-healing concrete, carbon-fiber-reinforced cement-based composites, and the application of nanotechnology in cement-based materials. This study mainly discusses the influence of nano-zinc oxide and nano-zirconia on cement-based materials. The addition of nano-zinc oxide improved the size change, setting time and compressive strength of cement [11]. Research has shown that the addition of nano-zinc oxide to calcium aluminate cement improves its dimensional change, setting time, and compressive strength.

The effects of nano-zinc oxide on the mechanical properties of cemented clayey sand and the microstructural properties of cemented clayey sand with nano-zinc oxide additive were investigated using high-resolution field-emission scanning electron microscopy (FESEM) by Abbas B et al. [12]. Their research results show that nano-zinc oxide had a positive effect on the mechanical properties, microstructure, and setting time of cement-based materials. Chen YZ et al. [13] conducted an experiment on Malaysian Portland cement mixed with nano-zirconia, and their analysis showed that its physical properties and bond strength were strengthened. The addition of nano-ZrO_2_ significantly enhances the mechanical properties of fracture resistance, compressive strength, diametral tensile strength, and micro-hardness of resin cement, as demonstrated by ElKemary Baraa M et al. [14]. Nano-zinc oxide and nano-zirconia had different surface effects, mechanical properties, and chemical activities. Therefore, the effects of the two on cement-based materials are also different.

There are still many gaps in the research on nanomaterials modified by cement-based materials. Most studies focus on common nanomaterials, such as nano-silica [15] and nano-titanium dioxide [16]. There are few studies on the exploration and application of new or special nanomaterials [17]. The deep mining and analysis of a large number of experimental data is not enough, and there is a lack of an effective mathematical model to accurately describe the quantitative relationship between the properties of nanomaterials and cement-based materials. In this study, nano-zinc oxide and nano-zirconia were used to replace the amount of cement under different substitution ratios to prepare specimens, and then, the mechanical properties, saturated water absorption, shrinkage properties, and thermogravimetric analysis of the cement specimens were tested. The influence mechanism of the two on cement-based materials was analyzed and compared from different angles.

## 2. Materials and Methods

### 2.1. Nanomaterials, Cement, and Sand

In this experiment, ordinary Portland cement (OPC, with a strength grade of 42.5R, according to Chinese national standard GB175-2023 [18]) was used as the binder. The test water was laboratory tap water. The test sand was from China Xiamen ISO Standard Sand Co., Ltd. (Xiamen, China), and the particle size distribution of the sand utilized was 0.08–2.0 mm, with a water absorption rate of 0.12%. Recently, the incorporation of nanomaterials in cement concrete composites has gained wide attention. [19] The nano-zinc oxide was from Guangzhou Metallurgy Co., Ltd. (Guangdong, China). The nano-zirconia used in the test was from Shanghai Yaoyi Alloy Materials Co., Ltd. (Shanghai, China). The physical parameters of the nanomaterials are shown in Table 1.

### 2.2. The Establishment of a Response Surface Model and the Production of the Specimens

#### 2.2.1. Establishment of Response Surface Model

**Figure 1:**
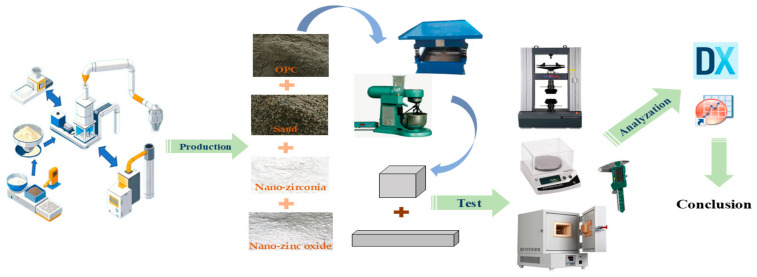
A flow chart of research.

The response surface method (RSM), an experimental technique to reduce the number of tests and the cost of research, was used to design the experiments and evaluate and optimize the results [12]. The type of nanomaterials, the amount of substitution, and the water–cement ratio in the mix ratio had a significant effect on the mechanical properties, coefficient of water saturation, autogenous shrinkage, dry shrinkage, and chemical composition of concrete. Based on the research of Lining W et al. [20] and Changsheng Gao et al. [21], the substitution amount of the nano-zinc oxide was set in the range of 0%~1%, and in order to obtain a larger range of influence law, this experiment was based on the research of Chen Yik Zhen et al. [13] and Behnam Behnia et al. [22], whereby the substitution amount of the nano-zirconia was set in the range of 0%~10%. In order to make the results of this study better, the water–cement ratio of high-performance concrete [23] and conventional concrete [24] was integrated. So, the water–cement ratio was set in the range of 0.2~0.4. The flow chart of research is shown in Figure 1.

In this experiment, the Design-expert 13 software was used to establish a mathematical model by the Box–Behnken experimental design (BBD) using a response surface analysis [25]. The test design involved three factors, three levels, and five central points, with the substitution amount of (A) nano-zinc oxide (%), the substitution amount of (B) nano-zirconia (%), and (C) the water–cement ratio as the influencing factors. The response values were 3 days of compressive strength (Y_1_), 28 days of compressive strength (Y_2_), 3 days of saturated water absorption (Y_3_), and 28 days of saturated water absorption (Y_4_). There were 17 groups of tests in the experiment, of which 12 groups were factorial tests, and five groups were repeated tests of the central point of the design area, which were used to detect the fitting of the central area and to determine the error of the tests. In addition, two groups of blank control group tests with a water–cement ratio of 0.2 and 0.3 were also conducted separately in this experiment. The test of each batch was repeated three times, and the results were averaged. The experimental design and results are shown in Table 2.

#### 2.2.2. Manufacturing of Specimens

For the cement mortar, the corresponding raw materials were weighed according to the mix ratio of the experimental design, and the specimens were prepared according to the requirements of the national industrial standard “Specification for mix proportion design of ordinary concrete” (JGJ 55-2011) [26]. The raw materials were fully mixed, and then, water was added. The mixer was used to fully stir evenly. The prepared mixture was then poured into the oiled molds with a size of 40 mm × 40 mm × 40 mm and 25 mm × 25 mm × 285 mm. The stirred cement mortar was loaded into the molds and moved to a cement mortar shaking table for vibration. Then, the surface of the cement mortar was smoothed with a scraper and covered with a plastic film. Finally, the molds were removed after resting at room temperature for 48 h. They were placed in a standard curing room (temperature: 20 ± 2 °C; relative humidity: 95% ± 5%) until they reached the age required for analysis. For the cement paste, according to the mix proportion of the experimental design, after weighing and mixing evenly, it was made into flakes and put into a standard curing room for curing to the required age.

### 2.3. Experimental Method

#### 2.3.1. Compressive Strength

The test used 40 mm × 40 mm × 40 mm cube test blocks that were cured for 3 days and 28 days under standard curing conditions, and each group measured the compressive strength of three cube samples. In this experiment, the YAW-300 B computer-controlled electro-hydraulic cement pressure testing machine produced by Jinan Times Gold Testing Machine Co., Ltd. (Jinan, China) was used to test the compressive strength of concrete blocks according to the requirements of the testing machine. According to the research of Wang Lining et al. [20] and Chen Yik Zhen et al. [13], the control mode was set to displacement control, and the test speed was 2mm/min to obtain a more accurate stress–strain curve.

#### 2.3.2. Shrinkage Performance

Molds with a size of 25 mm × 25 mm × 285 mm were used, and rivets were placed at both ends of the molds. After 48 h of casting, the samples were demolded. The self-shrinking specimens were wrapped with paraffin to isolate the external water vapor, and the dry-shrinking specimens were placed in a dry environment. Subsequently, the samples were placed horizontally on a laboratory bench, and the length and quality of the test pieces were measured regularly using an electronic vernier caliper and an electronic scale over a time span of 2 days to 28 days to minimize potential errors [27].

#### 2.3.3. Coefficient of Water Saturation

After 3 days and 28 days of standard curing, the specimens were taken out and placed in an oven at 65 ± 5 °C for 24 h. [28] The specimens were taken out and weighed. Then, the specimens were completely immersed in water at room temperature for 24 h until the saturated immersion weight was restored, and the cube test block was taken out and placed in a sieve. Then, the specimens were completely immersed in water for 24 h at room temperature until the saturated immersion weight was restored. The cube test block was taken out and placed in a sieve to drain until there were no water droplets in the lower part of the mortar specimens [29]. The formula used for saturated water absorption is as follows:

(1) $$\alpha =\frac{m_{w}-m_{d}}{m_{d}}\times 100\%$$

In this formula, $m_{w}$ is the mass of the test block in the wet state, and $m_{d}\;$ is the mass of the test block in the dry state.

#### 2.3.4. Contents of Calcium Hydroxide (CH) and Calcium Silicate Hydrate (CSH)

The weight loss interval was as follows: (1) The weight loss at 105 °C was attributed to the evaporation of free water. (2) The weight loss at 420 °C was due to weight loss caused by dehydration of the CSH gel phase. (3) The weight loss at 550 °C was due to CH dehydration. In this experiment, the contents of CSH and CH in the cement paste samples of ages of 3 days and 28 d were measured and evaluated. The samples of each group were put into an oven at 65 ± 5 °C for 10 h drying, and each group was divided into 3 parts, with an average of 10 ± 0.5 g per part. After this, all the samples were placed in an oven to bake at 105 °C, 420 °C, and 550 °C. At each temperature, samples were first dried for 1 h; and then, the oven was closed; and then, the samples were taken out and weighed and recorded after being kept at a constant temperature for 1 h [30,31]. The calculation formulas of the CSH and CH content are as follows:

(2) $$W_{CSH}=\frac{m_{105}-m_{420}}{m_{105}}\times 100\%$$

(3) $$W_{CH}=\frac{m_{420}-m_{550}}{m_{105}}\times 100\%$$

In this formula, $m_{105}$, $m_{420}$, and $m_{550}\;$ are the weight of the cement paste at 105 °C, 420 °C, and 550 °C.

## 3. Experimental Results and Analysis

In order to further explore the influence of nanomaterials on the enhancement of cement-based materials, the experimental performance and results in Table 2 were analyzed by response surface regression fitting using the Design-Expert13 software. In the process of a fitting analysis using the least squares method, the influence of a single factor and quadratic interactions between two factors on the response value was considered. The final fitting results show that the second-order model had the best fitting effect. The compressive strength and saturated water absorption of the cement mortar specimens with different ages designed in the experiment had the best quadratic relationship with nano-zinc oxide, nano-zirconia, and water–cement ratio. For the binomial fitting regression equations, see Formulas (4)–(7):

Compressive strength (3 days) Y_1_:

Y_1_ = 72.47 − 1.48 × A − 1.66 × B − 18.93 × C − 3.77 × AB + 1.6 × AC − 0.3261 × BC + 8.43 × A^2^ + 1.69 × B^2^ − 3.65 × C^2^ (4)

Compressive strength (28 days) Y_2_:

Y_2_ = 81.90 + 2.79 × A − 0.7331 × B − 18.5 × C+3.41 × AB − 1.18 × AC + 4.68 × BC − 1.59 × A^2^ + 3.97 × B^2^ − 0.5167 × C^2^ (5)

Coefficient of water saturation analysis (3 days) Y_3_:

Y_3_ = 4.96 + 0.2188 × A + 0.2075 × B + 2.08 × C − 0.1225 × AB + 0.12 × AC + 0.2475 × BC − 0.525 × A^2^ + 0.0325 × B^2^ − 0.095 × C^2^ (6)

Coefficient of water saturation analysis (28 days) Y_4_:

Y_4_ = 2.58 + 0.2675 × A + 0.2550 × B + 1.2 × C + 0.3900 × AB − 0.195 × AC − 0.325 × BC + 0.089 × A^2^ + 0.139 × B^2^ + 0.1090 × C^2^ (7)

To evaluate the accuracy of response surface prediction results, the f-value test and the *p*-value test can be used to judge the significance between an established model and response values. The *p*-value obtained from an analysis of variance (ANOVA) is used to examine the significance of each term. The lower the *p*-value, the greater the importance of each term of the model [32]. And the *p*-value test is used to determine the parameters of hypothesis testing and to evaluate the reliability of a fitted regression equation. A *p*-value less than 0.0500 indicates the model terms are significant. A *p*-value greater than 0.1000 indicates the model terms are not significant [12]. In Table 3, all the model *p*-values were less than 0.0500, indicating that the models were significant. All the lack-of-fit *p*-values of the models are greater than 0.0500, which implies that they are not significant relative to the pure error.

**Figure 2:**
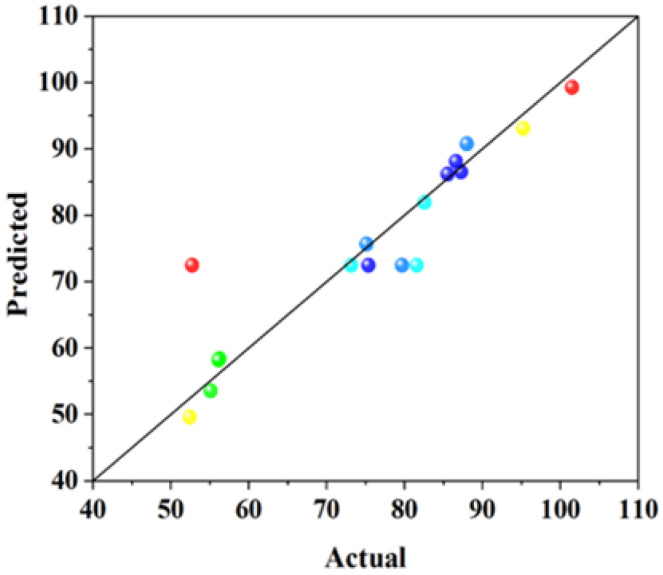
The relationship between the predicted values of the model and the actual values with 3 days of compressive strength.

**Figure 3:**
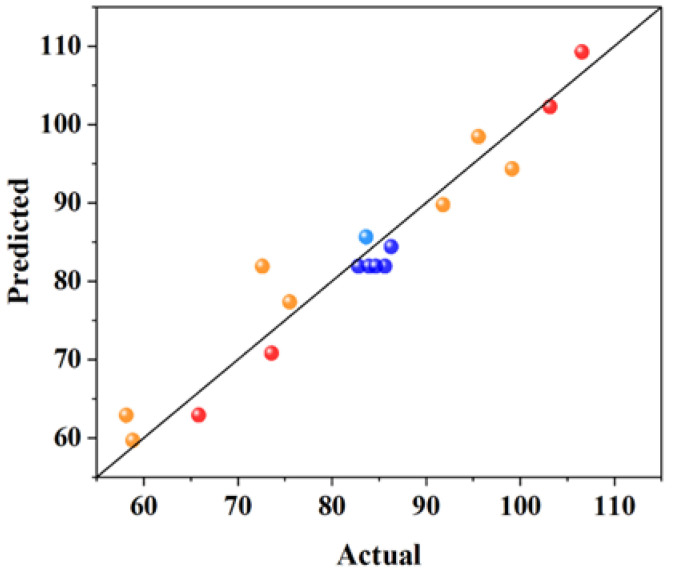
The relationship between the predicted values of the model and the actual values with 28 days of compressive strength.

**Figure 4:**
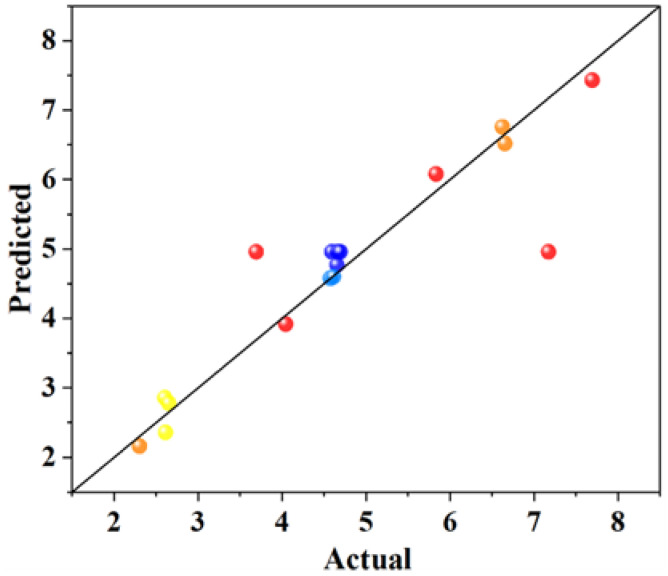
The relationship between the predicted values of the model and the actual values of the 3-day coefficient of water saturation.

**Figure 5:**
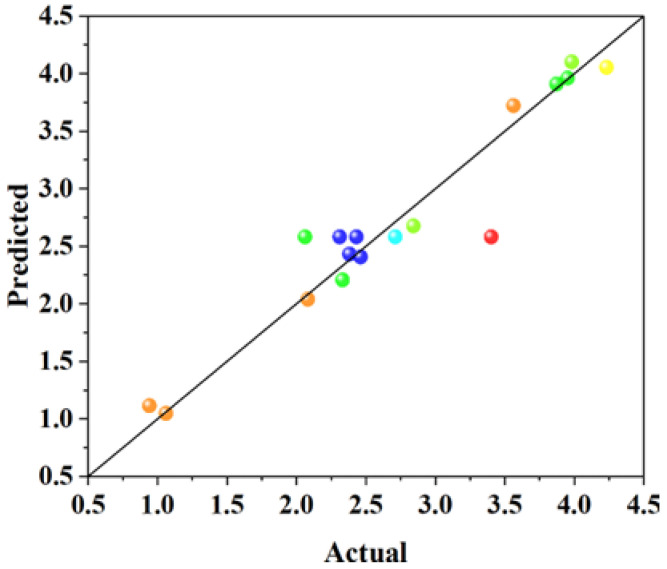
The relationship between the predicted values of the model and the actual values of the 28-day coefficient of water saturation.

It can be seen from Table 4 that the R^2^ of each group was 0.7440, 0.9357, 0.8375, and 0.9207, the adjusted R^2^ of each group was 0.6849, 0.8531, 0.6286, and 0.8187. In general, the closer R^2^ value is to 1, the smaller the error of the model is and the more accurate the regression equation is. It can be seen from the above analysis that all the regression equations are more accurate. The difference between the adjusted R^2^ and the predicted R^2^ in each group is small and less than 0.2 (adjusted R^2^; predicted R^2^ < 0.2), indicating that the prediction of the model is reliable. The Adeq precision values (the ratio of effective signal to noise) of each group are >4, indicating that the model is reasonable. Therefore, the four models are applicable to the prediction of related performance. The relationship between the predicted values of the response model and the experimental values is shown in Figure 2, Figure 3, Figure 4 and Figure 5. The actual measured values are represented by the *X* axis, and the predicted values of the model are represented by the Y axis. The correlation between the predicted values and the actual values is high. The actual measured values are evenly distributed on the straight line and its two sides, and the dispersion is small, indicating that the model has high fitting accuracy and good reliability.

### 3.1. Compressive Strength Analysis

**Figure 6:**
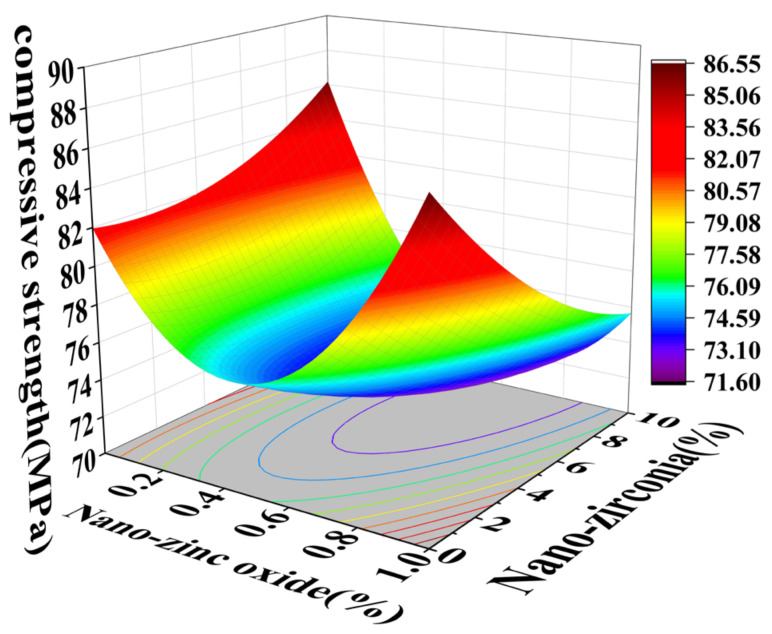
A 3D response surface diagram of the 3-day AB factor on compressive strength.

**Figure 7:**
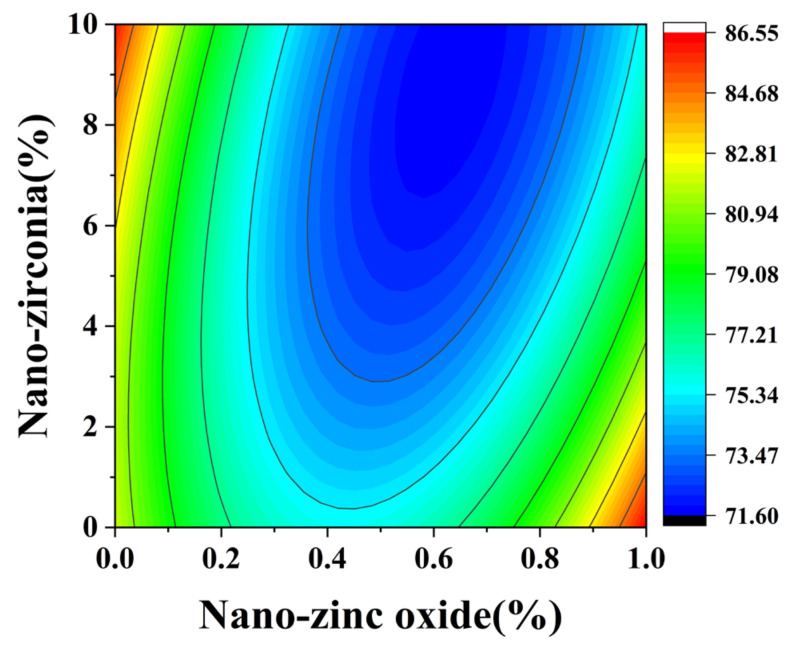
Contour map of the influence of the 3-day AB factor on compressive strength.

**Figure 8:**
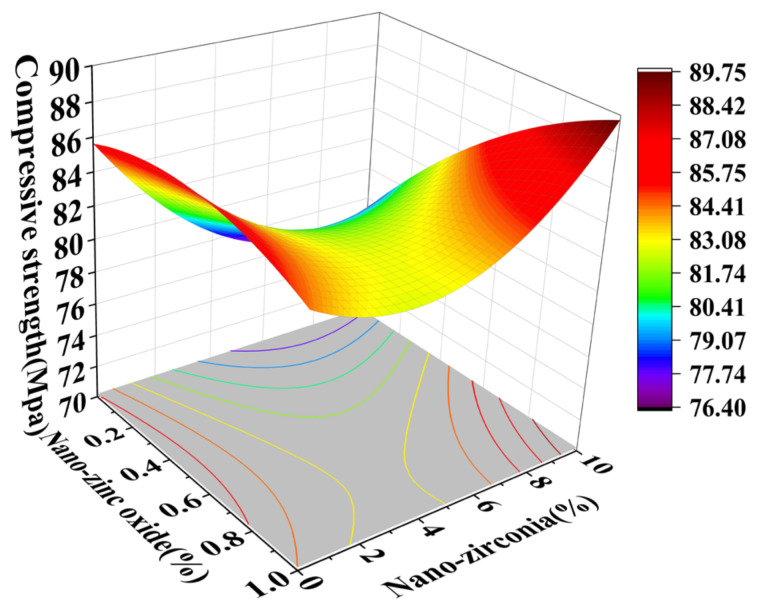
A 3D response surface diagram of the 28-day AB factor on compressive strength.

**Figure 9:**
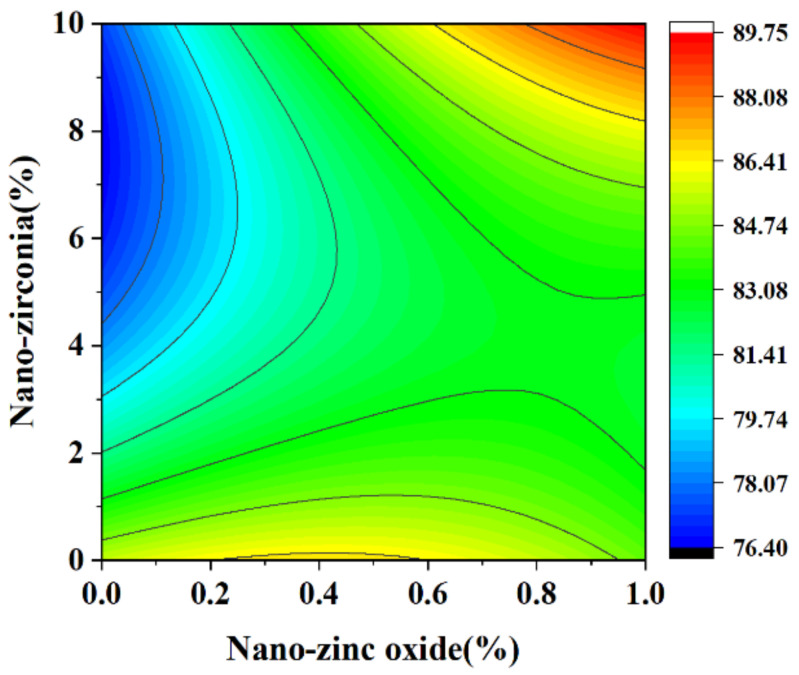
Contour map of the influence of the 28-day AB factor on compressive strength.

In the response surface diagram, the steeper the response surface slope is, the more significant the interaction between the two factors is and the greater the impact on the compressive strength of cement-based materials. Figure 6 and Figure 7 reflect the interaction between the substitution amount of nano-zinc oxide for cement (A) and the substitution amount of nano-zirconia for cement (B) at the age of 3 days on the compressive strength of the cement mortar specimens. It can be seen from Figure 7 that the contour map of the interaction between A and B is obviously oval, indicating that the interaction has a significant effect on the compressive strength of cement-based materials. Moreover, the radian of the response surface of A is greater than that of B, indicating that the influence of A is greater. Figure 8 and Figure 9 reflect the interaction between the substitution amount of nano-zinc oxide for cement (A) and the substitution amount of nano-zirconia for cement (B) at the age of 28 days on the compressive strength of the cement mortar specimens. It was observed that the compressive strength of the cement-based materials increased first and then decreased when the substitution amount of nano-zinc oxide to cement increased from low to high (0–1%). It was observed that the compressive strength of the cement-based materials first decreased and then increased with an increase in the substitution amount of nano-zirconia for cement from low to high (0–10%). In previous studies, the compressive strength of cement was shown to range from 6.2 MPa to 90.4 MPa [33]. Compared with this test, the compressive strength range is 52.3926 MPa~106.525 MPa, the lower limit of compressive strength increased by 88.17%, but the upper limit of compressive strength increased by 15.14% [34]. The compressive strength of the cement-based materials in this experiment also improved, indicating that the use of nanomaterials gives a uniform particle distribution, which results in a higher filler load and lowers viscosity, improving the compressive strength [13].

### 3.2. Coefficient of Water Saturation Analysis

**Figure 10:**
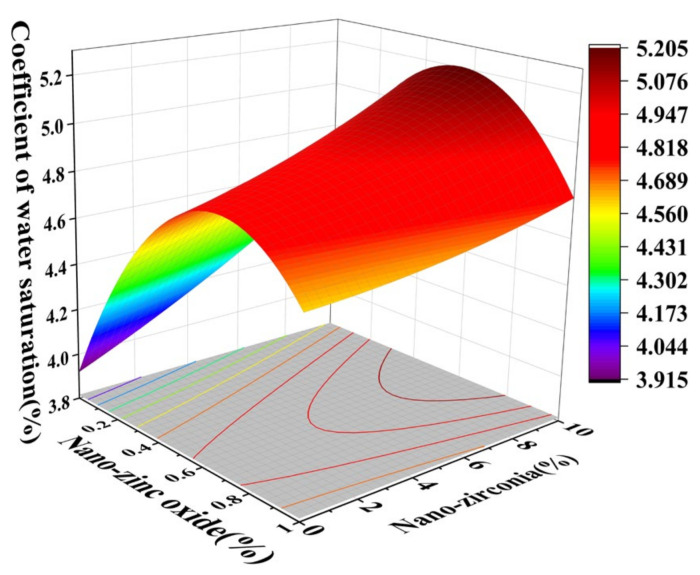
A 3D response surface diagram of the 3-day AB factor on the coefficient of water saturation.

**Figure 11:**
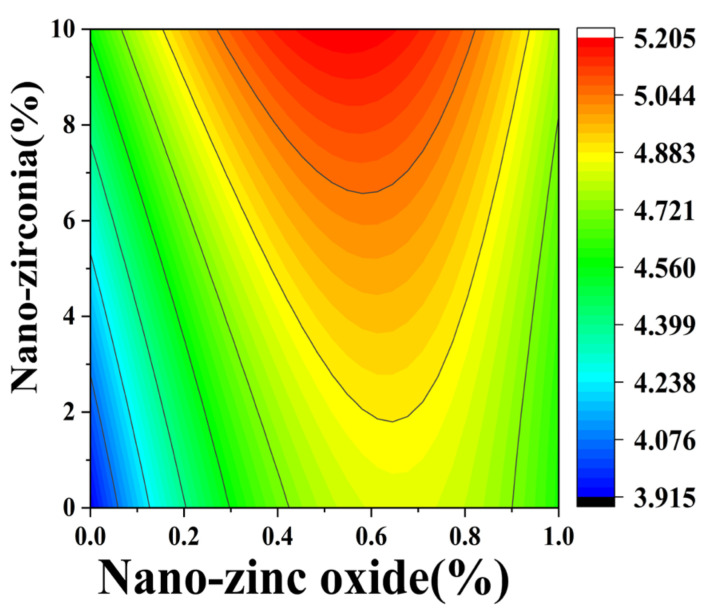
Contour map of the influence of the 3-day AB factor on the coefficient of water saturation.

**Figure 12:**
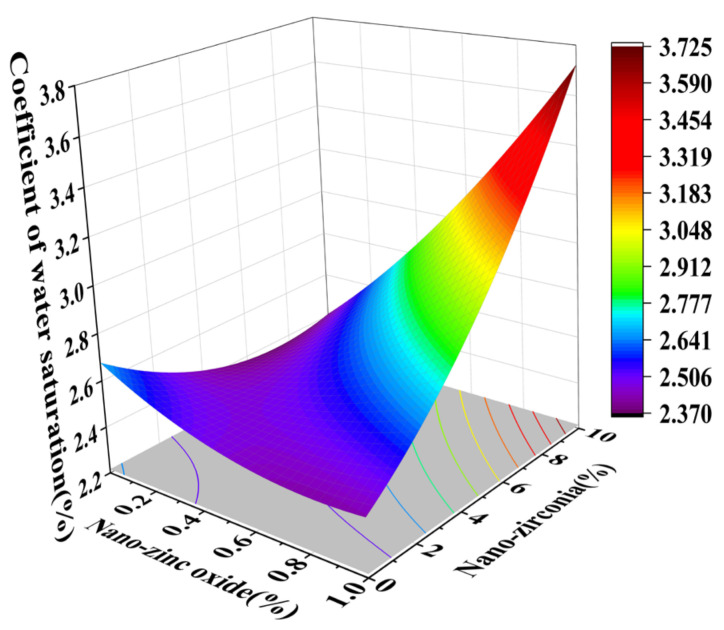
A 3D response surface diagram of the 28-day AB factor on the coefficient of water saturation.

**Figure 13:**
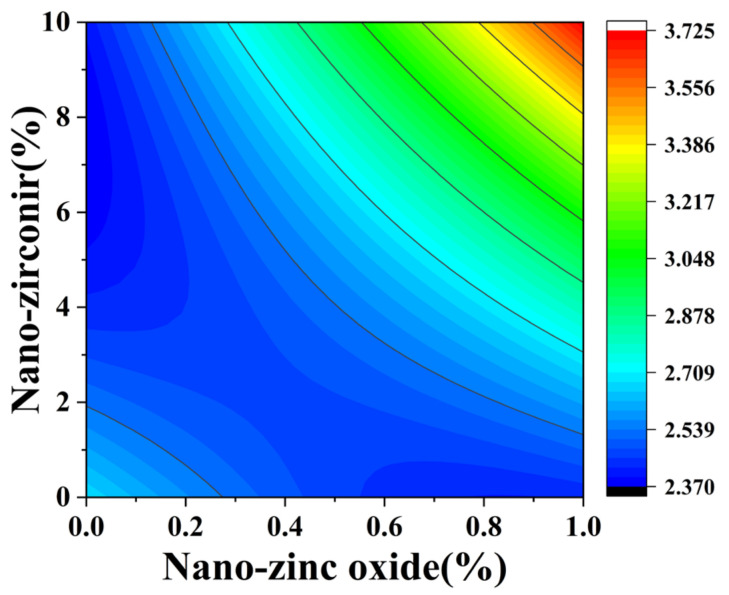
Contour map of the influence of the 28-day AB factor on the coefficient of water saturation.

Figure 10 and Figure 11 reflect the interaction between the substitution amount of nano-zinc oxide for cement (A) and the substitution amount of nano-zirconia for cement (B) at the age of 3 days on the coefficient of water saturation of the cement mortar specimens. It was observed that when the substitution amount of nano-zirconia was constant, the saturated water absorption of the cement specimens first increased and then decreased with an increase in the nano-zinc oxide substitution amount (0–1%). According to an analysis of the 2D contour map, the interaction between A and B was found to have a significant effect on the saturated water absorption of cement-based materials. Figure 12 and Figure 13 reflect the interaction between the substitution amount of nano-zinc oxide for cement (A) and the substitution amount of nano-zirconia for cement (B) at the age of 28 days on the coefficient of water saturation of the cement mortar specimens. It can be seen that when the substitution amount of nano-zirconia is 0%, with an increase in the substitution amount of nano-zinc oxide, the saturation water absorption response surface radian of the 28-day age is smaller. It is shown that the effect of nano-zinc oxide on the saturated absorption rate of the cement-based materials is not significant. Compared with the saturated absorption rate of an age of 3 days, the saturated absorption rate of the 28-day age decreased. When the water–cement ratio was 0.4, it decreased by 2.69%. When the water–cement ratio was 0.3, it decreased by 2.06%. When the water–cement ratio was 0.2, it decreased by 0.94%. When the water–cement ratio was 0.2, the saturated water absorption rate of the 3-day age and 28-day age was smaller than that of the water–cement ratio of 0.3 and 0.4. Therefore, the lower water–cement ratio in this experiment could have reduced the saturated water absorption of the cement materials. Overall, the water absorption and porosity for all the mixtures complied with the requirement limit of 18% for masonry construction [35]. Single-doped nanomaterials cause cement-based materials to have a dense microstructure and low capillary action and improve the compactness of concrete by refining pores. Therefore, the use of nano-composite materials resulted in a reduced water absorption of the cementitious compounds [19].

### 3.3. Shrinkage Performance Analysis

**Figure 14:**
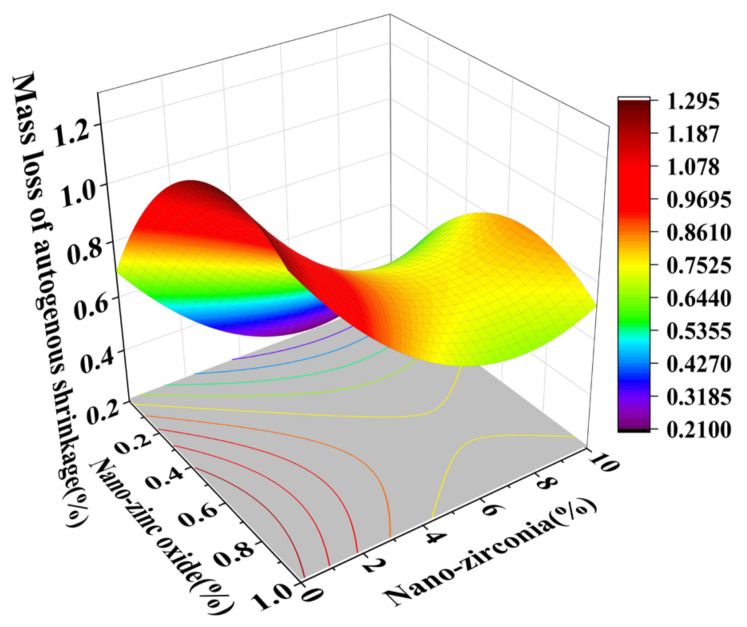
A 3D response surface diagram of the AB factor on the mass loss of autogenous shrinkage.

**Figure 15:**
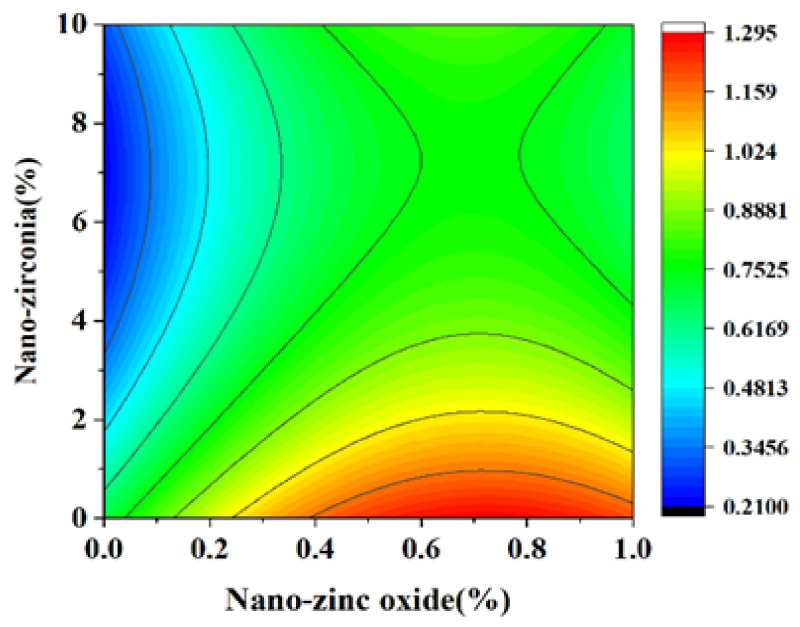
Contour map of the influence of the AB factor on the autogenous shrinkage of mass loss.

**Figure 16:**
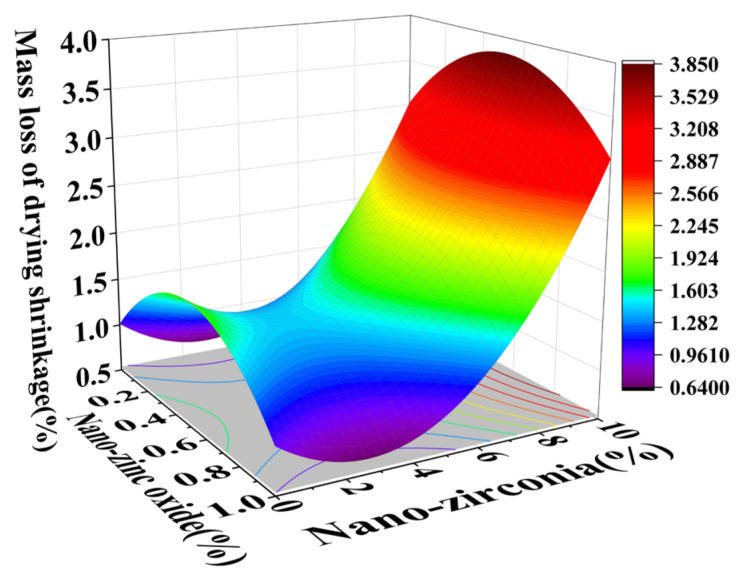
A 3D response surface diagram of the AB factor on the mass loss of drying shrinkage.

**Figure 17:**
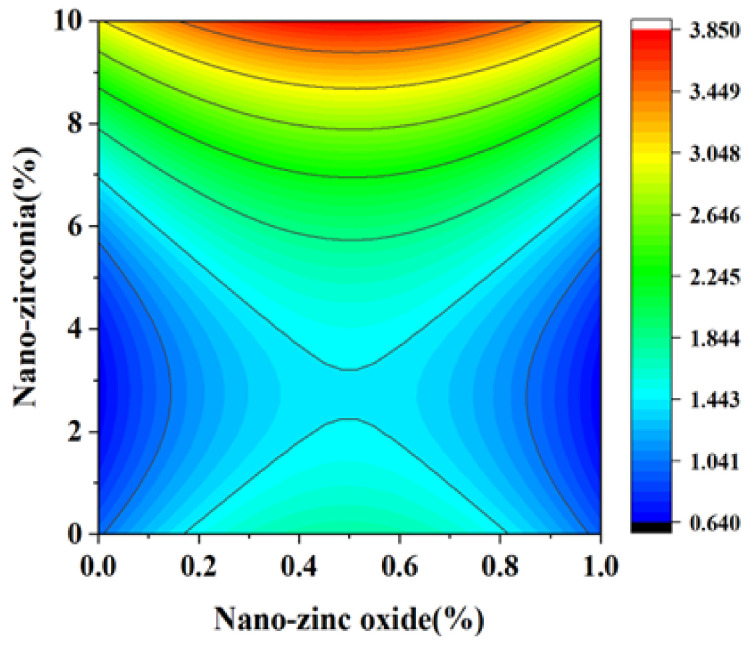
Contour map of the influence of the AB factor on the drying shrinkage of mass loss.

**Figure 18:**
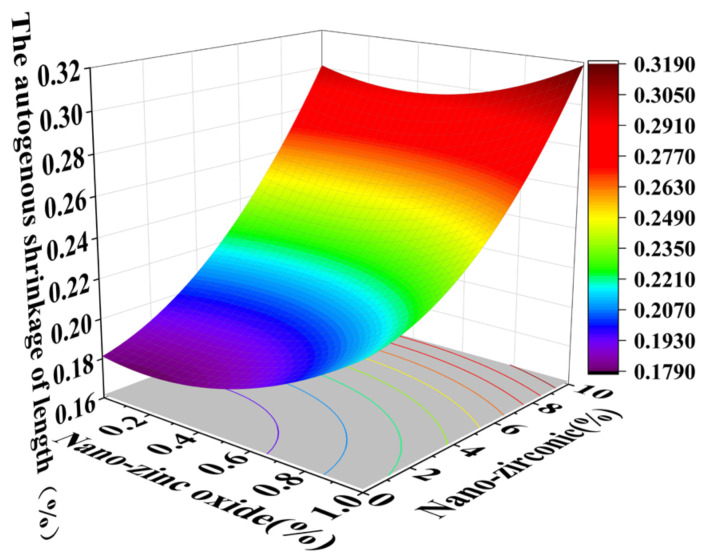
A 3D response surface diagram of the AB factor on the autogenous shrinkage of length.

**Figure 19:**
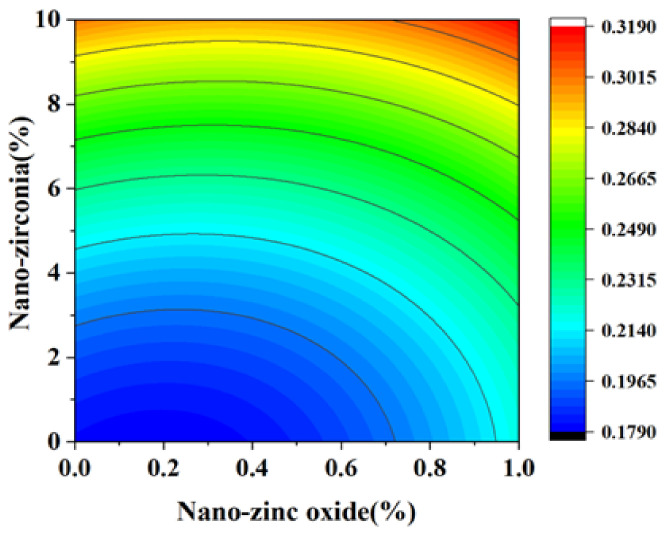
Contour map of the influence of the AB factor on the autogenous shrinkage of length.

**Figure 20:**
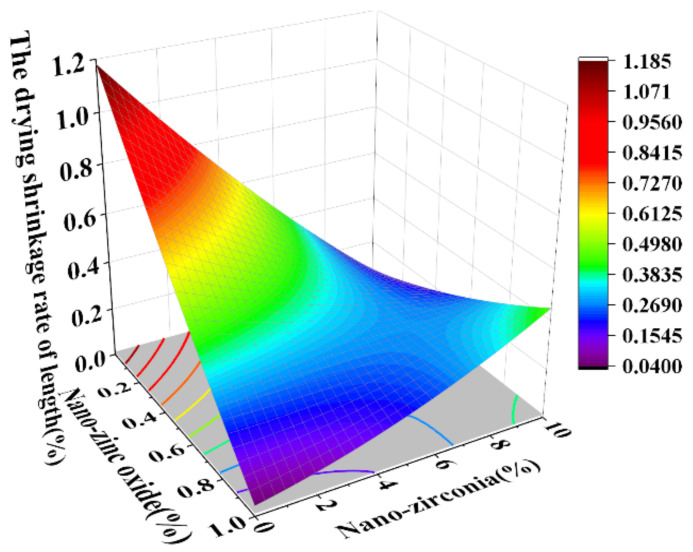
A 3D response surface diagram of the influence of the AB factor on the drying shrinkage rate of length.

**Figure 21:**
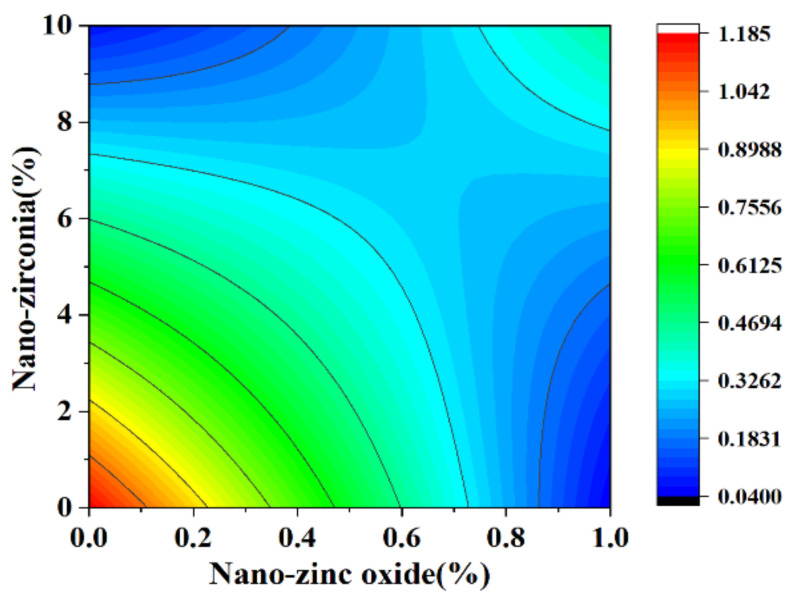
Contour map of the influence of AB factor on the drying shrinkage rate of length.

Shrinkage properties can be divided into two categories: drying shrinkage and autogenous shrinkage [36]. Concrete cracking induced by shrinkage has always been considered to be a major concern of concrete durability and structural safety control [37]. Autogenous shrinkage refers to the reduction in the macroscopic volume of a system caused by the hydration reaction of the material without water exchange with the environment. Drying shrinkage refers to the shrinkage or length changes of hardened concrete due to the loss of capillary water [38]. This loss of water can lead to capillary tension in the pore structure, which may lead to the formation of cracks [39]. Figure 14, Figure 15, Figure 16 and Figure 17 are the response surface diagrams and the corresponding contour change diagrams of the effects of the substitution amount of nano-zinc oxide for cement (A) and the substitution amount of nano-zirconia for cement (B) on the mass loss of autogenous shrinkage and the mass loss of drying shrinkage of the cement specimens. It was concluded that the increase in nano-zinc oxide (0–1%) first increased the mass loss of the cement-based materials and then decreased it. With an increase in the nano-zirconia (0%~10%) content, the mass loss of the cement-based materials first decreased and then increased. According to the different radians of the response surface, it could be concluded that nano-zirconia had the most significant effect on the mass loss of drying shrinkage. This shows that a small amount of nano-zinc oxide substitution accelerates the loss of cement quality, while a small amount of nano-zirconia substitution reduces the loss of quality. The research of Xiaoyan Liu et al. shows that nanomaterials can be positive additives for cement-based materials for improving the microstructures and restricting the autogenous shrinkage of cement-based materials [37]. Therefore, the degree of mass loss is affected by the amount and type of substitution of nanomaterials. A substitution amount of 0.4–0.8% nano-zinc oxide had the greatest influence on the mass loss. It can be seen from Figure 18, Figure 19, Figure 20 and Figure 21 that the substitution amount of nano-zirconia had a significant effect on the length of self-shrinkage, and the substitution amount of nano-zinc oxide had little effect. Nano-zinc oxide had a great influence on the length of drying shrinkage, while the substitution amount of nano-zirconia had little effect. Therefore, nanomaterials have different effects on the shrinkage of length in different environments.

### 3.4. The Content of Calcium Hydroxide (CH) and Calcium Silicate Hydrate (CSH)

**Figure 22:**
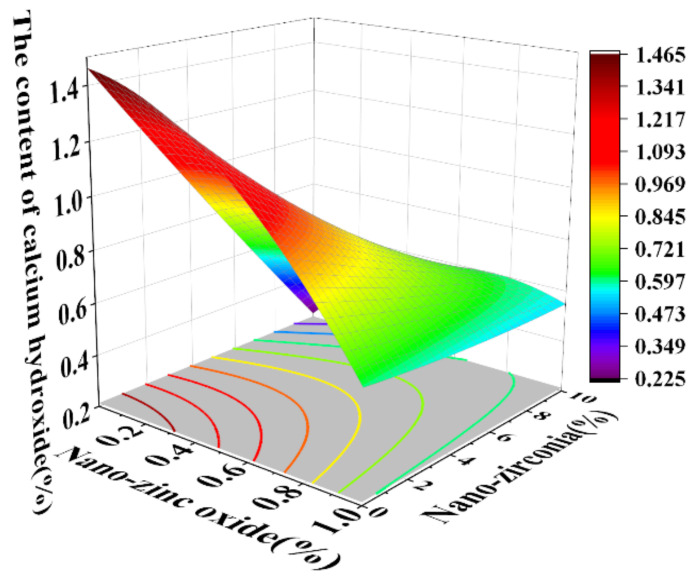
A 3D response surface diagram of the 3d AB factor on the content of calcium hydroxide.

**Figure 23:**
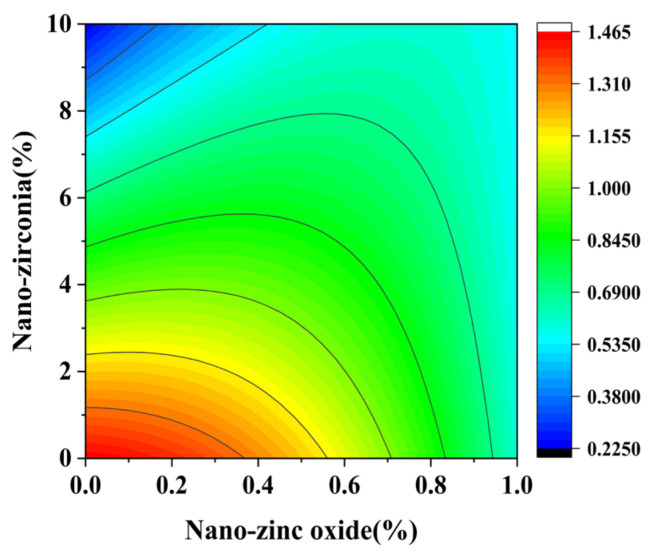
Contour map of the influence of the 3d AB factor on the content of calcium hydroxide.

**Figure 24:**
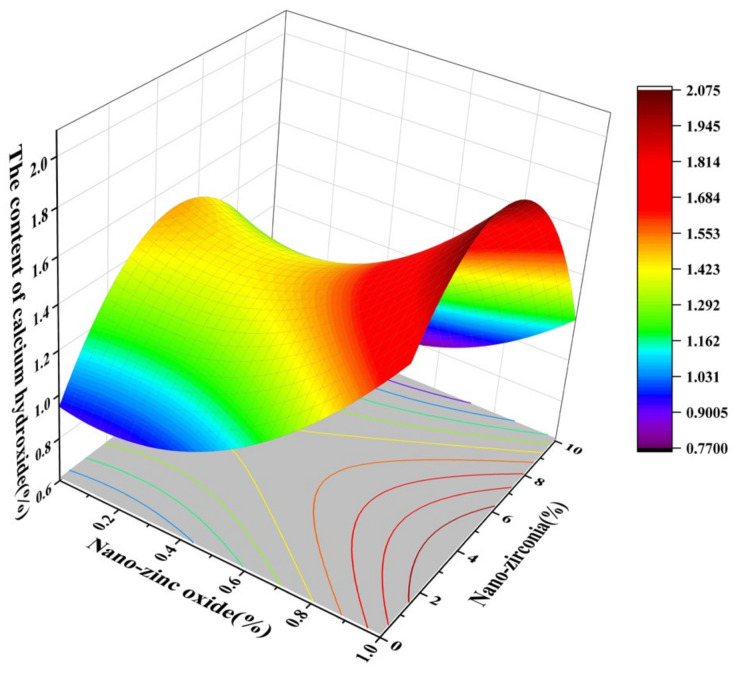
AB factor on the content of calcium hydroxide.

**Figure 25:**
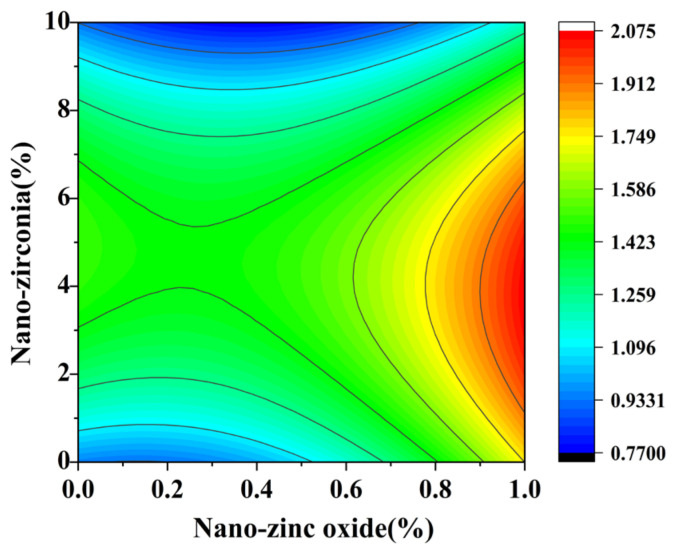
Contour map of the influence of the 28d AB factor on the content of calcium hydroxide.

CH is the second most abundant hydration product after CSH in cement hydration products. CH tends to form a unique hexagonal diamond crystal, and its morphology may be affected by space, temperature, and other substances in the system during the growth process [40,41]. Studying the effects of nanomaterials on the content of CH helps to understand their influence on the microstructure and structure of cement slurry [42]. It can be seen from Figure 22 and Figure 23 that nano-zinc oxide has a great influence on the content of CH. With an increase in nano-zinc oxide (0%~1%), the content of CH decreases gradually. This shows that nano-zinc oxide promotes the reaction of cement-based material with CH. This proves that the combination of nano-zinc oxide can effectively limit the content of CH. Therefore, the hardness and roughness of CSH are improved, and the structural defects are reduced. This result is confirmed by the works by Abbas B et al. [12] and Han et al. [43], which investigate the efficiency of inactive nanoparticles. It can be seen from Figure 24 and Figure 25 that when the age is 28 days, the substitution amount of nano-zirconia has a greater influence on the content of CH. With an increase in the nano-zirconia substitution amount (from 0% to 10%), the content of CH first increases and then decreases. The CH content is closely related to the degree of cement hydration, and a higher CH content corresponds to a larger degree of hydration [44]. Therefore, the substitution amount of nano-zirconia is 4–6%, and the hydration degree is higher. The degree of hydration is reduced if the substitution amount is too low or too high. This may be attributed to the fact that the number of zirconia nanoparticles present in the mix was more than the dosage required to combine with the liberated lime during the process of determining the hydration degree. At a higher dosage of nano-zirconia, for their high surface area, agglomeration occurred, which caused the hydration degree to weaken.

**Figure 26:**
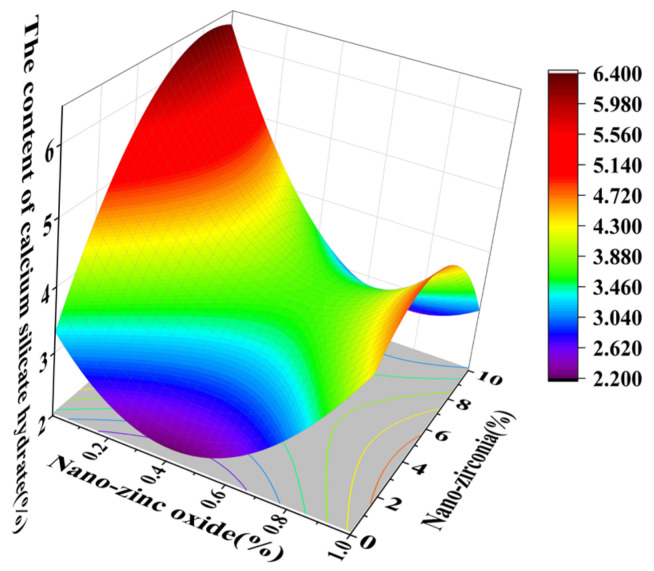
The 3D response surface diagram of the 3-day AB factor on the content of calcium silicate hydroxide.

**Figure 27:**
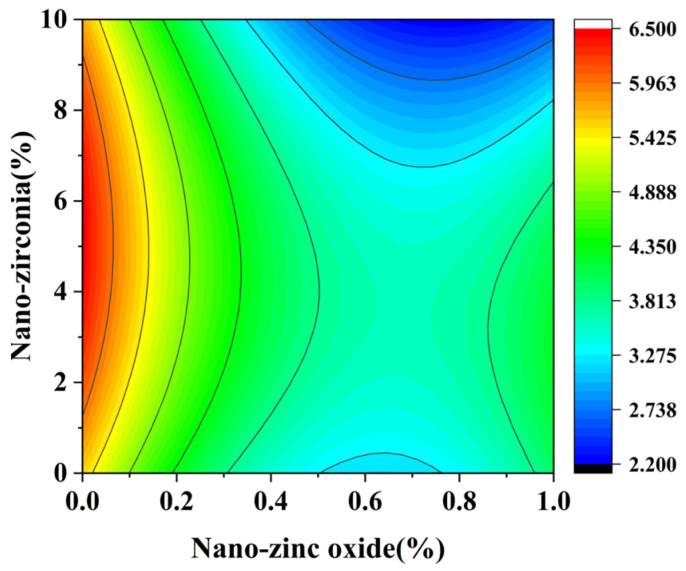
Contour map of the influence of the 3-day AB factor on the content of calcium silicate hydroxide.

**Figure 28:**
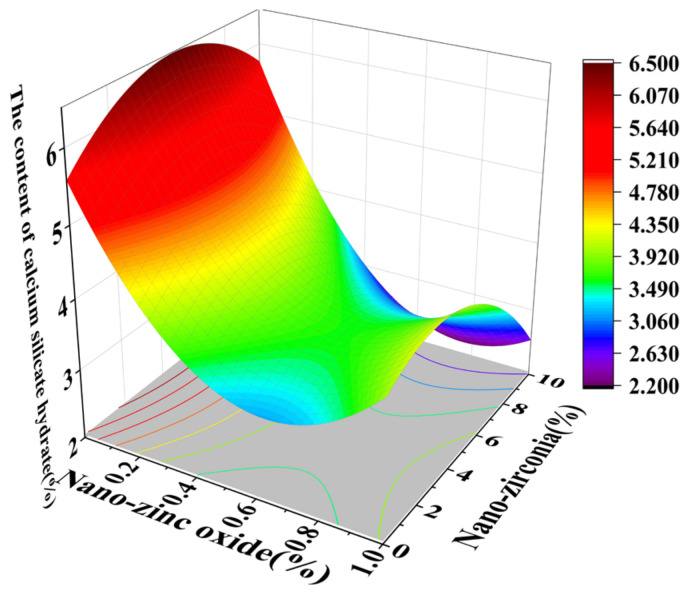
The 3D response surface diagram of the 28-day AB factor on the content of calcium silicate hydrate.

**Figure 29:**
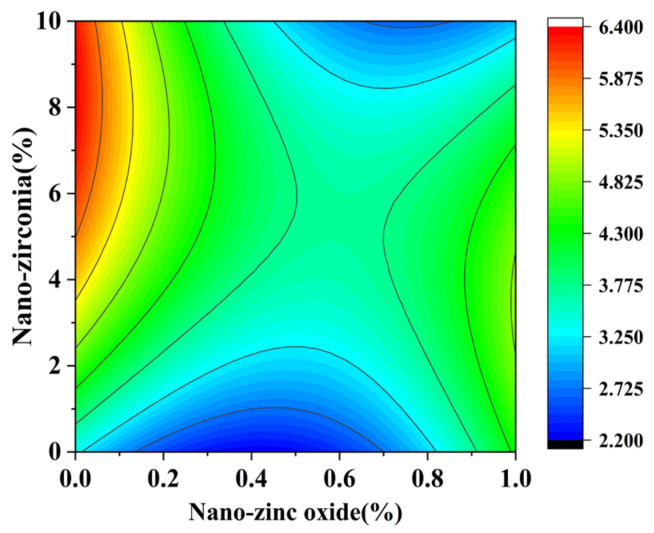
Contour map of the influence of the 28-day AB factor on the content of calcium silicate hydrate.

Nano-silica (NS) can react with crystalline-phase calcium hydroxide (CH) to generate amorphous CSH gels [45]. CSH is used to promote the early strength growth of cement stone. The dispersion of nanomaterials is crucial for fully realizing their properties [46]. More CSH results in a densified microstructure, contributing to an enhancement in the strength and durability of the cementitious materials [42]. Figure 26, Figure 27, Figure 28 and Figure 29 show the effects of the interaction between the amount of nano-zinc oxide substitution (A) and the amount of nano-zirconia substitution (B) on the content of CSH at the age of 3 days and 28 days. Through observation and analysis, it was found that the CSH content of 3 days and 28 days first decreased and then increased with an increase in the nano-zinc oxide substitution amount (0% to 1%). With an increase in the substitution amount of nano-zirconia (0% to 10%), it first increased and then decreased. As shown in the research by Han et al. [44], non-pozzolanic nano-fillers can accelerate the formation of CSH in cementitious samples. The content of CSH is the lowest in the range of 0.4–0.6% of nano-zinc oxide, and the highest in the range of 4–6% of nano-zirconia. Previous studies have shown that nanoscale CSH constitutes 60–70% of the volume of the cement stone, exerting critical influence on the mechanical and durability properties of cementitious materials [47], and CSH is considered a source of strength in cement-based materials [48]. Therefore, the compressive strength of cement is the lowest when the substitution amount of nano-zinc oxide is 0.4% to 0.6%. However, the effect of CH on the micromechanical properties of CSH remains debatable. Some studies have shown that CH fills the pores of CSH and leads to improved mechanical properties of CSH, enhancing the overall mechanical performance, thereby improving the mechanical properties of cementitious materials [20,41]. Therefore, the content of CH increases in the later stage to improve the mechanical properties of cement-based materials.

## 4. Conclusions

This study effectively proves the feasibility of adding two different nanomaterials to cement-based materials as partial cement substitutes. This study provides new insights into the mechanical properties, the coefficient of water saturation, and shrinkage properties of cement mortar using nanomaterials to replace part of cement, as well as the evaluation and analysis of the CH content and CSH content of cement paste. The effects of nano-zinc oxide and nano-zirconia on the properties of cement-based materials were analyzed and compared.

The three factors have significant effects on compressive strength, the coefficient of water saturation, and the mass loss of autogenous shrinkage. Nano-zinc oxide and nano-zirconia replace part of cement, which increases the lower limit of compressive strength of cement-based materials by 88.17% and the upper limit by 15.14%. The content of nano-zirconia plays a key role in cement-based materials, and its content has a significant effect on the mass loss of autogenous shrinkage and drying shrinkage. The dispersion of nano-zinc oxide in cement-based materials is related to chemical reaction activity. Nano-zirconia can effectively promote the hydration reaction of cement, thereby improving the density and strength of cement-based materials. Partial replacement of cement with nanomaterials can not only significantly improve the performance of cement-based materials but also reduce the amount of cement, thereby reducing carbon emissions. This provides a new basis for the sustainable development of cement-based materials. The incorporation of nanomaterials can optimize the microstructure of cement-based materials and can improve their mechanical properties and durability.

## Figures and Tables

**Table 1:** Physical parameters of nanomaterials.

Name	Mean Diameter (nm)	Purity (%)	Specific Surface Area (m^2^/g)	Volume Density (g/cm^3^)	Density (g/cm^3^)	Crystal Form	Color
Nano-zinc oxide	20	99.99	100	0.41	5.6	Globular	White
Nano-zirconia	20	99.99	95	1.98	6.0	Monoclinic	White

**Table 2:** Experimental design and results.

Serial Number	Influence Factor			Response Value			
	A	B	C	Y_1_/MPa	Y_2_/MPa	Y_3_/%	Y_4_/%
1	0	−1	1	55.0842	65.8202	6.65	3.98
2	0	0	0	75.3317	85.6096	7.17	2.43
3	0	1	−1	86.5781	95.5268	2.65	2.33
4	1	0	1	56.2102	58.123	6.62	4.23
5	−1	1	0	85.5009	75.484	4.57	2.46
6	0	0	0	79.65	72.5788	3.69	2.06
7	0	0	0	73.119	82.7936	4.59	2.71
8	1	−1	0	87.2233	86.2618	4.61	2.38
9	0	0	0	81.5281	83.9072	4.69	2.31
10	−1	−1	0	82.5387	83.5948	4.04	2.84
11	−1	0	1	56.0825	58.8089	5.83	3.87
12	0	1	1	52.3926	73.5458	7.69	3.95
13	1	1	0	75.0862	91.7801	4.65	3.56
14	0	−1	−1	87.9653	106.525	2.6	1.06
15	−1	0	−1	101.475	99.1047	2.3	0.94
16	0	0	0	52.7044	84.6178	4.66	3.4
17	1	0	−1	95.198	103.138	2.61	2.08

**Table 3:** ANOVA for quadratic model.

Source	Y_1_		Y_2_		Y_3_		Y_4_	
	F	P	F	P	F	P	F	P
Model	4.52	0.0297	11.32	0.0021	4.01	0.0403	9.03	0.0042
A	0.2152	0.6568	2.10	0.1906	0.3745	0.5599	3.32	0.1114
B	0.2680	0.6207	0.1451	0.7145	0.3369	0.5798	3.01	0.1261
C	34.99	0.0006	92.41	<0.0001	33.81	0.0007	67.02	<0.0001
AB	0.6955	0.4318	1.57	0.2508	0.0587	0.8155	3.52	0.1025
AC	0.1251	0.7340	0.1880	0.6777	0.0563	0.8192	0.8812	0.3791
BC	0.0052	0.9446	2.96	0.1291	0.2397	0.6394	2.45	0.1617
A^2^	3.65	0.0977	0.3597	0.5676	1.14	0.3220	0.1932	0.6735
B^2^	0.1471	0.7127	2.24	0.1782	0.0044	0.9493	0.4713	0.5145
C^2^	0.6859	0.4349	0.0379	0.8511	0.0372	0.8526	0.2898	0.6070
Lack of Fit	0.1019	0.9547	1.12	0.4411	0.0705	0.9727	0.1938	0.8958

**Table 4:** Fit statistics.

Response Value	R^2^	Adjusted R^2^	Predicted R^2^	Adeq Precision	C.V./%
Y1	0.7440	0.6849	0.6071	9.6776	11.62
Y2	0.9357	0.8531	0.4766	11.8772	6.58
Y3	0.8375	0.6286	0.6283	6.7942	21.59
Y4	0.9207	0.8187	0.7310	9.5878	15.16

## Data Availability

The original contributions presented in this study are included in the article, and further inquiries can be directed to the corresponding author.
